# Clouclip combined with a questionnaire on the influence factors of myopia in children

**DOI:** 10.3389/fped.2023.1228257

**Published:** 2023-08-07

**Authors:** Ya Zhang, Ming Su, Yanhua Sun, Liqin Qi, Lifang Gao, Xueya Wu, Yutong Li, Yanli Liu, Wei Li, Minxiao Jin

**Affiliations:** Department of Optometry, Shijiazhuang Aier Eye Hospital, Shijiazhuang, China

**Keywords:** clouclip, questionnaire, myopia, near work, children, influence factors

## Abstract

**Purpose:**

To evaluate eye use behavior in myopic and non-myopic children objectively using Clouclip M2 device and subjectively using questionnaire and compare the results. The study also aimed to assess the relationships between ocular biometric parameters and refractive status.

**Methods:**

Clouclip M2 was used in monitoring eye use behavior and visual environment in children aged 9–11 years. The participants were monitored for 7 days. On the eighth day, data stored in the device were collected, relevant eye examination were conducted and survey questionnaire was administered. The paired sample *t*-test was used to compare the eye use behavior obtained objectively and subjectively. The relationships between ocular biometric parameters and refractive status were assessed using the Pearson's Correlation analysis.

**Results:**

Spherical equivalent refraction was significantly correlated with axial length, axial length to corneal radius, anterior chamber depth, lens thickness, and corneal radius (*P* < 0.05). The average time per day spent on near work, the maximum time for single near work, and the average near working distance were significantly lower, and the average total time spent on outdoor activities was significantly longer as determined by questionnaire method than that found using Clouclip M2. Logistic regression analysis revealed that prolonged near work, shorter working distance, presence of parental myopia, and lesser outdoor activities were significant risk factors for myopia.

**Conclusions:**

The childhood myopia is influenced by eye use behavior, eye use environment, and parental myopia. Results from this study further support that biometric and optical parameters of the eye determine refractive status. Being an objective method, Clouclip M2 provides an independent eye use behavior data which potentially are more reliable than obtained from subjective method. Our study provided a theoretical basis for myopia prevention and control in clinical practice.

## Introduction

Ten years ago, myopia affected approximately 1.5 billion individuals worldwide, with a prevalence rate of 22% (1). The worldwide surge in myopia has led to a projected estimation of 4.7 billion individuals by 2050 (2). Myopia has emerged as a global public health concern in recent years, especially in China and other East Asian countries. In China, the prevalence of myopia among children aged 7–12 increased from 25.3% in 2008 to 32.8% in 2022 (3), with the highest incidence noted among primary school children (4).

Ocular refraction is dependent on optical and biometric parameters of the eye including curvatures of anterior and posterior corneal and lenticular surfaces, axial thicknesses of cornea, anterior chamber, lens and vitreous chamber, and refractive indices of ocular media (cornea, aqueous, lens and vitreous). Predominantly, change in ocular refraction occurs during the early childhood which is primarily brought about by the coordinated growth of its refractive components, including corneal and lens refractive power (Km, LP), anterior chamber depth (ACD), lens thickness (LT), and axial length (AL) (5). The changes in ocular refraction may also be influenced by eye use behaviors and environments such as exposure to outdoor illumination. As the epidemiology of myopia has increasingly focused on risk factors (6), researchers have identified increased near work and/or decreased time spent outdoors as possible factors contributing to its onset (7, 8). The majority of the previous studies investigating myopia-related environmental factors quantified through questionnaires, often leading to inaccurate information due to recall bias (9). More recently, investigators have used technologically more advanced electronic devices to monitor eye use behaviors objectively. The Clouclip M2 is one such wearable device that monitors the eye use behavior and its environment, such as durations and distances of near work, time spent on outdoor activity, and luminance of the working environment. Since accurate usage characteristics are vital to arrive at proper conclusions, and objective method like this could provide more reliable parameters that subjective questionnairs which are dependent on memory, cannot provide.

This study used both objective (using Clouclip M2 device) and subjective (using survey questionnaire) methods to determine eye use behavior and visual environment in children with a view to assess if these differ between myopic and non-myopic individuals. The results would be worthwhile in identifying the factors influencing myopia development and provide a theoretical basis for prevention and control of myopia. Additionally, we also investigated the relationship between optical parameters of the eye (AL, Km, LT, ACD, etc.) and refractive status.

## Subjects and methods

The development and implementation of this study was approved by the Ethics Committee of Aier Eye Hospital, Shijiazhuang, and all procedures followed the principles of the Declaration of Helsinki. Informed consents were sought and obtained from the students and their parents voluntarily prior to administering study related eye examinations. Clouclip M2 monitoring device was issued after obtaining informed consent from the school principal, homeroom teacher, along with students, and parents.

Students of Caochang Street Primary School (Shijiazhuang, Hebei province, China) were screened for eligibility. The inclusion criteria were age between 9 and 11 years, willing to consent to participate, willing to wear the device during the entire study period, be able to standardizing Clouclip M2 usage, no apparent abnormalities; be able to respond survey questionnaire and attend follow-up examinations.

Students with previous history of amblyopia, eye trauma, or eye pathology other than refractive error, general or mental illness, undergoing local or systemic drug use affecting test results, not sleeping between 24:00 and 6:00, undergoing orthokeratology, low-concentration atropine eye drops, repeated low-level red-light, or visual training, and unable or unwilling to comply with the Clouclip wearing requirements were excluded from the study. Data from the students not responding to survey questionnaire, not returning the Clouclip prior to 7 days of wear and not returning for eye evaluations on 8th day were not included in the analysis.

A total 257 primary school students aged between 9 and 11 years screened and consented to participate in the study. All participants were instructed to wear Clouclip M2 for 7 consecutive days, including 5 working days and 2 rest days. On the 8th day, accompanied by their parents, the students returned the Clouclip M2 and underwent relevant eye examinations and completed the survey questionnaire. Data from Clouclip M2 was then retrieved and analysed.

Among the 257 students, 39 had incomplete data collected by the clouclip M2, 4 could not cooperate to complete the questionnaire, and 2 withdrew from the study midway. Remaining 212 (105 boys and 107 girls) were included in the statistical analysis in final.

### Clouclip M2: basic principles and wearing requirements

**Figure 1:**
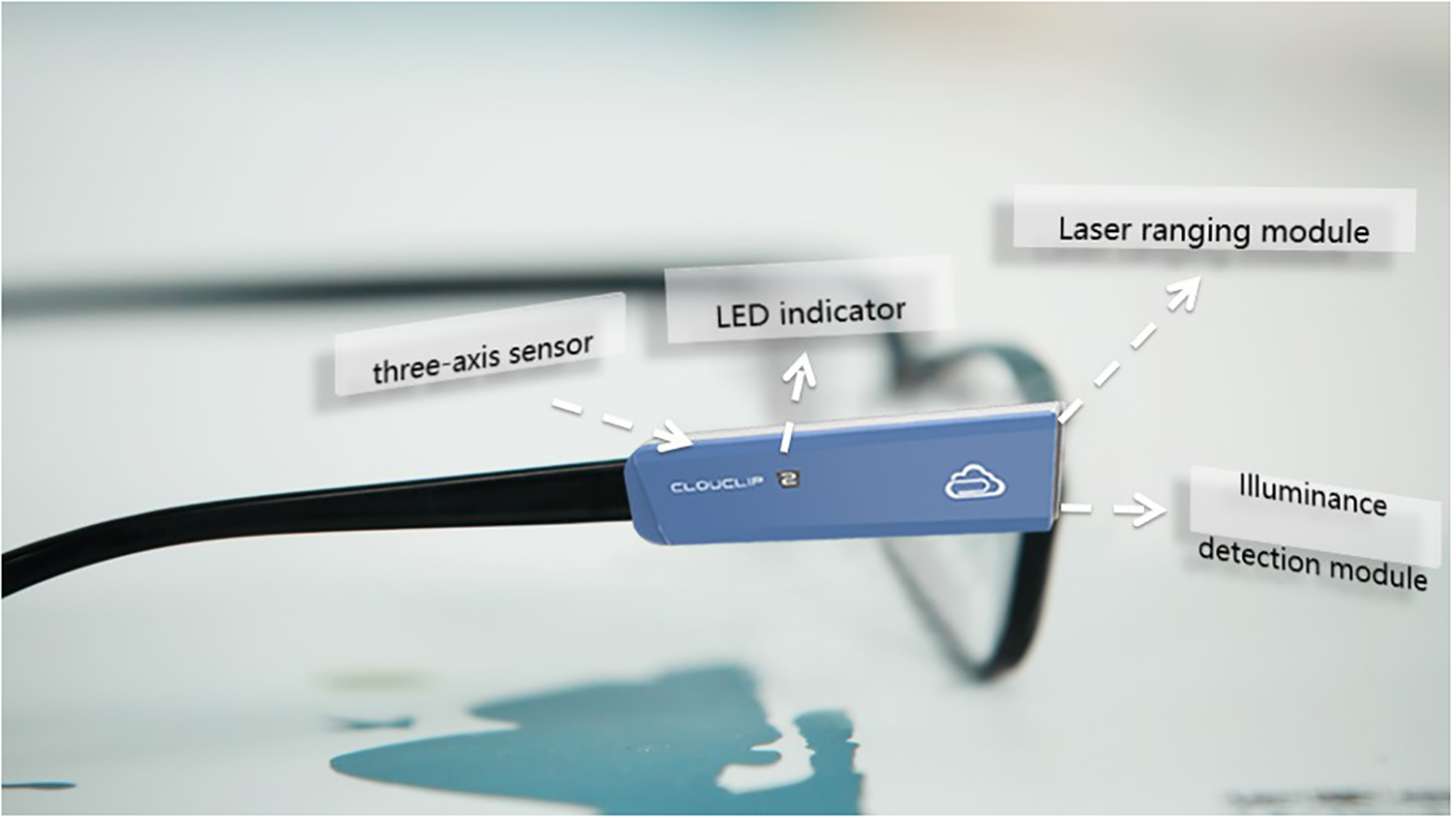


The Clouclip M2 (Figure 1) is designed to clip on the right arm of spectacle frame. The device is equipped with infrared sensors for viewing distance and luminance, and a three-axis accelerometer. The infrared tracking beam is emitted from the vertex, roughly aligned with the direction of the visual axis. The viewing distance is calculated by the time difference between transmitting and receiving the infrared beam. The distance of 60 cm or closer is considered as close working distance. The luminance sensor measures ambient luminance. Outdoor exposure is considered if luminance of the viewing surface exceeds 800 lux for more than 2 min. The default internal system of Clouclip M2 uses 6:00–18:00 as the daytime time and 18:00–24:00 as the evening.

Before the issuance, investigators thoroughly demonstrated the wearing requirements, techniques and precautions of the Clouclip M2 to the students and homeroom teachers, as applicable. The lensless frame was provided for those not wearing glasses. Specifically, instruction on the Clouclip M2 to be fixed parallel to the leg of the glasses to ensure front end of the device flush with the curved surface of the spectacle lens. The participants were required to wear the device continuously during non-sleep periods, remove it before going to bed, recharge it when not worn (during sleep), and wear it as they wake up next morning. Teachers and parents were responsible for monitoring the Clouclip M2 wear at school and home respectively.

**Figure 2:**
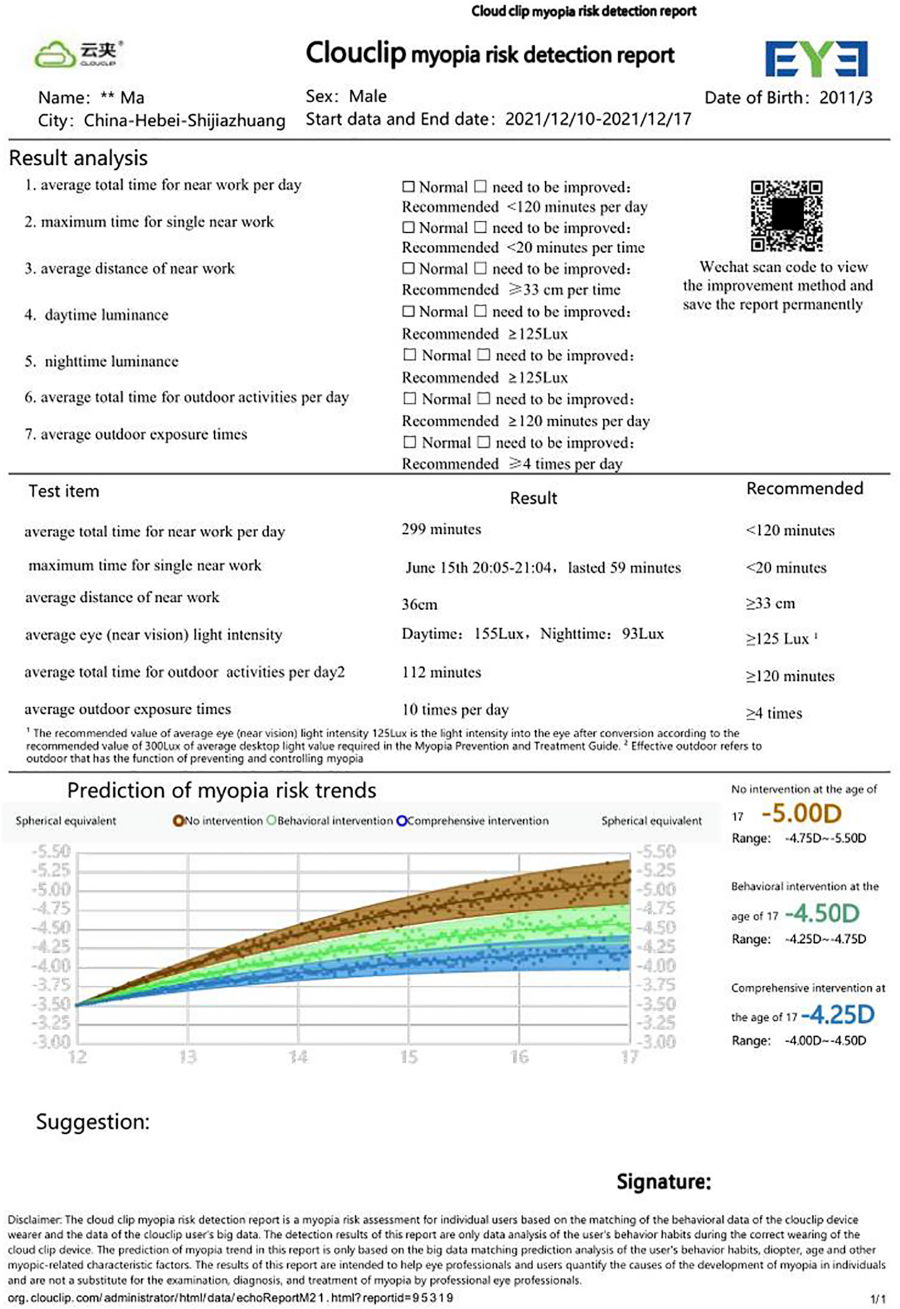


For the study purpose, seven parameters we noted from the device readings, which included the average total time for near work per day, the maximum time for single near work, the average distance for near work, the average total time for outdoor, the average outdoor exposure duration, daytime luminance and nighttime luminance (Figure 2).

### Refractive examination, ocular biometry, anterior segment and fundus examination

Objective refraction (without cycloplegia) was performed using an automated computerized optometer (AR-1, NIDEK, Japan) 3 times consecutively for consistency of measurement. A difference of ≤0.50 D in spherical or cylindrical components between the measurements was considered valid. Additional measurements were obtained for any inconsistency in the measurement. IOL Master700 (Carl Zeiss, Germany) was used to measure AL, Km, LT, and ACD. The image with the highest signal-to-noise ratio was selected for 3 consecutive measurements. The slit lamp (TOPCON SL-D4) and direct ophthalmoscope (Suzhou 66 vision YZ6f) were used to examine the anterior segment and fundus, respectively.

Spherical Equivalent (SE) refraction was calculated as measured by the autorefractor (SE = Sphere + ½ Cylinder). Myopia was defied as SE ≤ −0.50 D in either or both eyes, emmetropia as −0.50 D < SE < +0.50 D in both eyes and hypermetropia as SE ≥ +0.50 D. The non-myopic group in this study consisted of emmetropic and hypermetropic subjects.

### Questionaire

**Figure 3:**
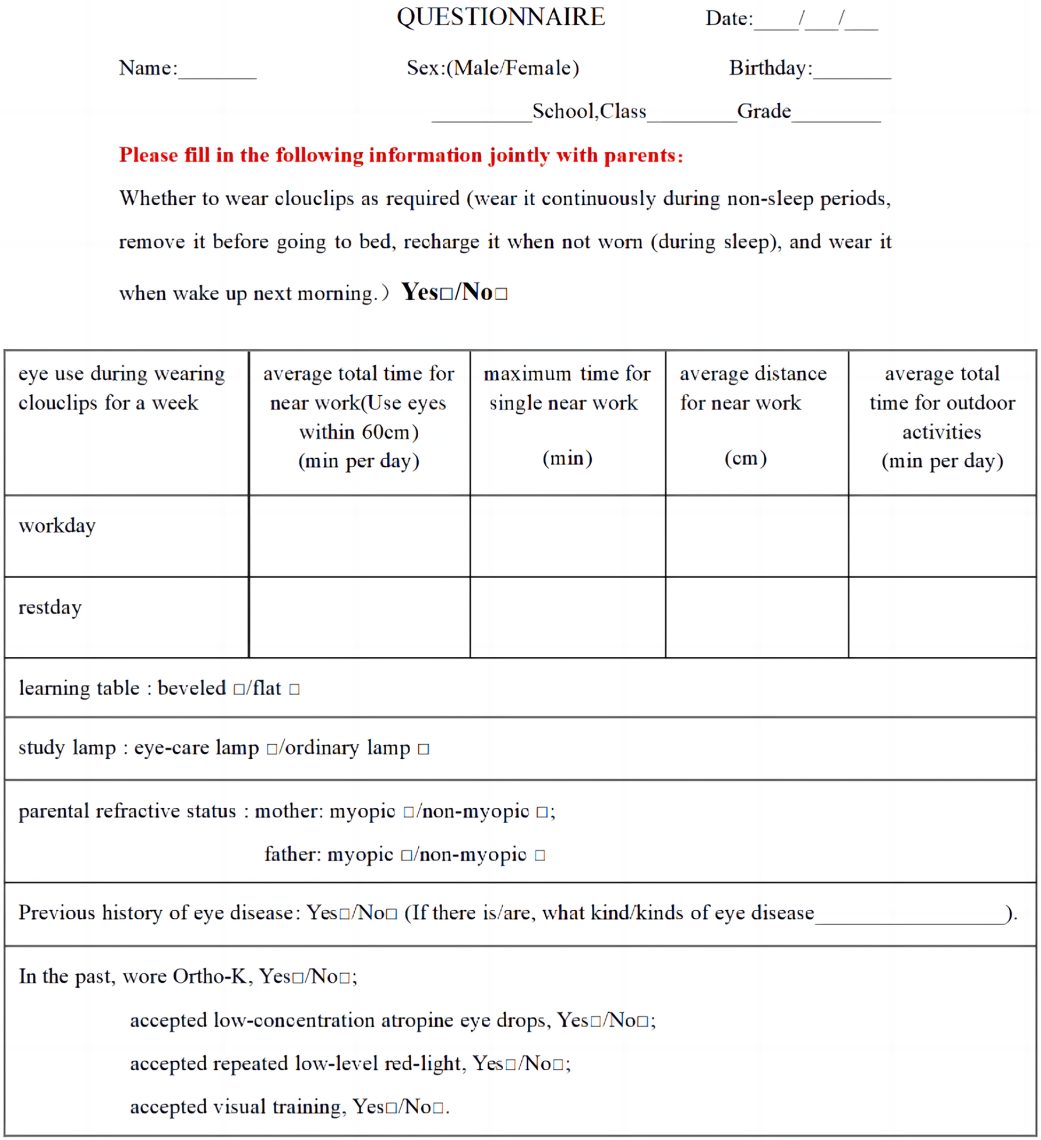


Subjective evaluation of eye use behaviours and environment involved administration of questionnaire that has been used previously in National Student Physique and Health Survey 2019 (10). The items in the questionnaire were formulated according to the Clouclip M2 project. The questionnaire contains items for the maximum time spent on single near work, the average distance of near work, and the average outdoor exposure duration. Additional items include information on whether the students habitually use flat or bevelled desk, use of standard or eye-care lamp for studying, have any myopic parents (both, one or none) (Figure 3). The average daily eye use time or exposure duration was calculated as below:

$$\mathrm{Average}\;\mathrm{duration}=\frac{5\times (\mathrm{time}\;\mathrm{during}\;\mathrm{working}\;\mathrm{days})+2\times (\mathrm{time}\;\mathrm{during}\;\mathrm{rest}\;\mathrm{days})}{7}.$$

### Statistics

SPSS (version 25.0) was used for statistical analysis. Central tendency was expressed as mean ± standard deviation after conforming for normal distribution of the data. Outcome variables obtained objectively using Clouclip M2 and subjectively from survey questionnaire were compared using paired sample *t*-test. Pearson correlation analysis was used to analyze the correlation between SE and ocular biometric parameters. The chi-square test was used for univariate analysis of eye use behavior and myopia where statistically significant variables in the univariate analysis of Clouclip M2 and questionnaire were taken as independent variables, and the regression model was established by stepwise backward binary logistic regression analysis. Statistical difference of *P* < 0.05 was considered significant.

## Results

Data from a total of 212 students (105 males and 107 females) were analysed, out of which, 162 students were myopic, 47 were emmetropic and 3 (only females) were hyperopic (Table 1).

**Table 1:** Number of the children tested.

	9 years		10 years		11 years		Total
	Boys	Girls	Boys	Girls	Boys	Girls	
Myopia	23	27	31	31	26	24	157
Emmetropia	9	7	9	8	7	6	55
Hyperope	0	3	0	0	0	0	0
Total	32	37	40	39	33	30	212

### Correlation between biometric parameters and Se refraction

**Figure 4:**
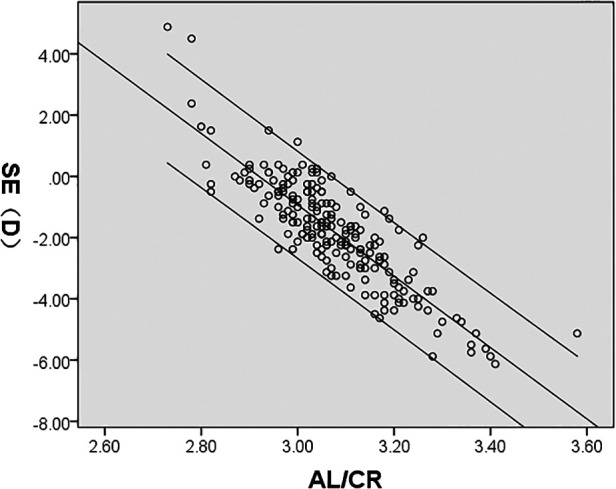


**Figure 5:**
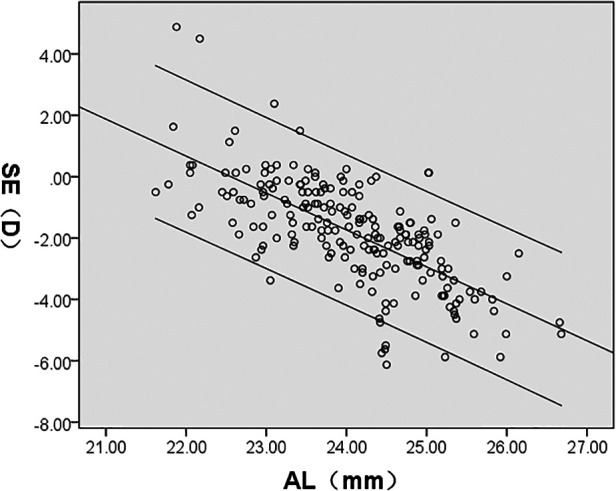


**Figure 6:**
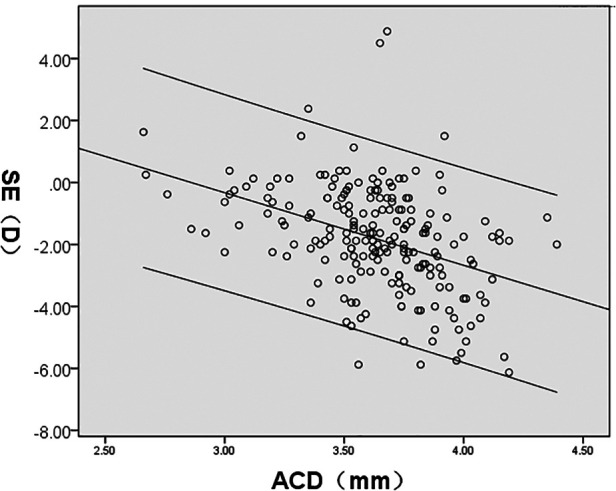


**Figure 7:**
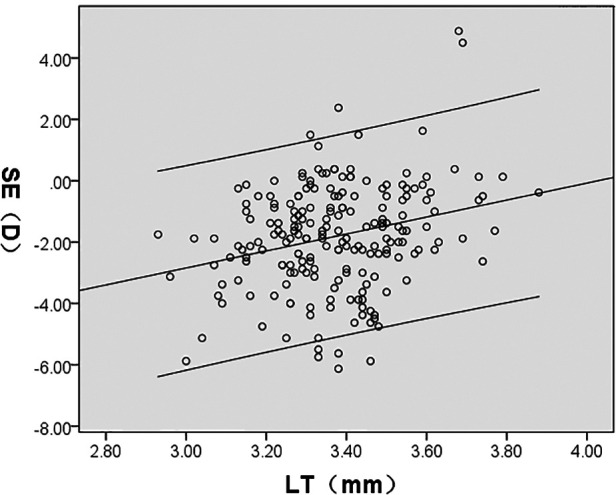


**Figure 8:**
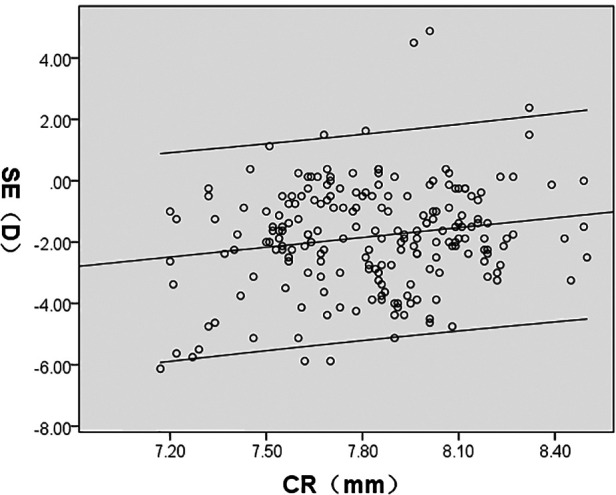


SE was significantly correlated with AL/CR, AL, ACD, LT, and CR (*P* < 0.05); AL/CR, AL, and ACD had a negative correlation whereas LT and CR demonstrated a positive correlation with SE. However, CCT had no significant correlation with SE (*P* > 0.05) (Figures 4–8, Table 2).

**Table 2:** Correlation analysis between eye biological parameters and SE.

		AL/CR	AL (mm)	ACD (mm)	LT (mm)	CR (mm)	CCT (µm)
SE(D)	*r*	−0.859	−0.695	−0.400	0.264	0.176	0.074
	*P*	<0.001	<0.001	<0.001	<0.001	0.012	0.291

*P* value indicates whether SE is correlated with AL/CR, AL, ACD, LT, CR and CCT; SE, spherical equivalent; AL, axial length; CR, corneal curvature radius, ACD, anterior chamber depth; LT, lens thickness; CCT, central corneal thickness.

### Comparison of eye use behaviour and environment data: Clouclip M2 vs. survey questionnaire

As measured by Clouclip M2, the average total time for near work per day was 294.80 ± 109.114 min, the maximum time for single near work was 81.56 ± 37.54 min, the average near working distance was 32.21 ± 4.44 cm, and the average total time for outdoor activities per day was 61.50 ± 27.97 min compared to 250.10 ± 103.10 min, 40.88 ± 18.37 min, 24.94 ± 8.04 cm, and 73.24 ± 35.75 min, respectively as determined using the survey questionnaire. A statistically significant differences occurred for the average total time for near work per day, the maximum time for single near work, the average distance for near work, and the average total time for outdoor activities per day (*P* < 0.05). In particular, the questionnaire survey showed a significantly lower average time spent at near activities, the maximum time for single near work, and the average near working distance, and longer average time spent on outdoor activities (Table 3).

**Table 3:** Clouclip M2 and the average value of eye behavior and eye environment data measured in questionnaire survey.

	Clouclip M2	Questionnaire survey	*t*	*P*
Average total time for near work per day (min)	294.80 ± 109.114	250.10 ± 103.10	3.243	0.001
Maximum time for single near work (min)	81.56 ± 37.54	40.88 ± 28.37	6.087	<0.001
Average distance of near work (cm)	32.21 ± 4.44	24.94 ± 8.04	12.394	<0.001
Average total time for outdoor activities per day (min)	61.50 ± 27.97	73.24 ± 35.75	−4.174	<0.001
Average outdoor exposure times per day (times)	7.91 ± 4.78	–	–	–
Daytime luminance (lux)	181.96 ± 63.41	–	–	–
Nighttime luminance (lux)	88.25 ± 55.97	–	–	–

*P* value indicates whether there is statistical significance in the difference between cloud clip M2 and the relevant contents of the questionnaire.

### Univariate analysis of eye use behavior eye environment

Clouclip M2 results showed statistically significant differences between the myopic and non-myopic groups in all aspects of eye use behaviors: (proportion of the number of people whether the average total time for near work per day reached 180 min, whether the maximum time for single near work reached 60 min, whether the average distance of near work reached 30 cm, whether the average total time for outdoor activities per day reached 90 min, whether the average number for outdoor activities per day reached 8 times) and eye use environment (whether the luminance for daytime use was 200 lux and for nighttime use was 125 lux) (*P* < 0.05) (Table 4).

**Table 4:** Univariate analysis of Clouclip M2 eye behavior, eye environment, and screening myopia in students.

	Clouclip M2			
	Myopia	Non-myopia	*Χ* ^2^	*P*
Average total time for near work per day				
<180 min	19	13		
≥180 min	143	37	6.072	0.014
Maximum time for single near work				
<60 min	42	23		
≥60 min	120	27	7.242	0.007
Average distance of near work				
<30 cm	52	15		
≥30 cm	110	35	0.078	0.780
Average total time for outdoor activities per day				
<90 min	118	26		
≥90 min	44	24	7.616	0.006
Average outdoor exposure times				
<8 times	78	14		
≥8 times	84	36	6.314	0.012
Daytime luminance				
<200 lux	96	21		
≥200 lux	66	29	4.602	0.032
Nighttime luminance				
<125 lux	104	24		
≥125 lux	58	26	4.190	0.041

*P* value indicates whether there is statistical difference in the behavior of each eye measured by Clouclip M2 between the myopia group and the non-myopia group.

### Single-factor analysis of ocular behavior and parental myopia obtained from the questionnaire survey

The questionnaire survey results showed statistically significant differences between the myopic and non-myopic groups in eye use behavior (proportion of the number of people whether the average total time for near work per day was 180 min, whether the maximum time for single near work was 60 min, whether the average distance of near work was 30 cm, and whether the average total time for outdoor activities per day was 90 min) and eye use environment (whether the study lamp was used, whether the parents had myopia) (*P* < 0.05), while no statistical significance in whether the beveled learning table was used (*P* > 0.05) (Table 5).

**Table 5:** Single-factor analysis of ocular behavior and parental myopia obtained from the questionnaire survey and screening myopia of students.

	Questionnaire survey			
	Myopia	Non-myopia	*X* ^2^	*P*
Average total time for near work per day				
<180 min	39	22		
≥180 min	123	28	7.402	0.007
Maximum time for single near work				
<60 min	139	44		
≥60 min	23	6	39.649	<0.001
Average distance of near work				
<30 cm	115	26		
≥30 cm	47	24	6.184	0.013
Average total time for outdoor activities per day				
<90 min	123	29		
≥90 min	39	21	6.050	0.014
Learning table				
Beveled	19	7		
Flat	143	43	0.183	0.669
Study lamp				
Eye-care lamp	128	31		
Ordinary lamp	34	19	5.898	0.015
Parents				
No myopic parent	24	16		
One myopic parent	74	26		
Two myopic parents	64	8	12.520	0.002

*P* value indicates whether there is statistical difference in the behavior of each eye in the nearsighted group and non-nearsighted group.

### Binary multi-factor logistic regression analysis of Clouclip M2 measured eye use behavior, eye environment

The prediction accuracy of this model was 77.8%.

The probability of children being myopic with an average total time of near work per day duration ≥180 min was 3.24 times higher than those with an average total time of near work per day <180 min (*P* < 0.05). Students with an average total time for outdoor activities per day <90 min were 2.44 times more likely to become myopic than those spending ≥90 min. Students exposed to <200 lux daytime luminance were 2.04 times more likely to develop myopia than those exposed to luminance ≥200 lux (*P* < 0.05) (Table 6).

**Table 6:** Binary multi-factor logistic regression analysis of Clouclip M2 measured eye behavior, eye environment, and myopia of students.

Independent variable	OR (95% CI)	*P*
Average total time for near work per day		
<180 min	1.00	
≥180 min	3.24 (1.40–7.52)	0.006
Average total time for outdoor activities per day		
≥90 min	1.00	
<90 min	2.44 (1.23–4.82)	0.010
Daytime luminance		
≥200 lux	1.00	
<200 lux	2.04 (1.03–4.01)	0.040

*P* value represents the binary multi-factor logistic regression analysis of Clouclip M2 measured eye behavior and student myopia.

### Binary multi-factor logistic regression of eye use behavior and parents’ myopia and students’ screening myopia obtained from the questionnaire survey

The probabilities of being myopic with an average total time of near work per day ≥180 min was 3.12 times higher than those with an average total time of near work per day <180 min (*P* < 0.05). Likewise, students were 2.61 times more likely to become myopic when they spend <90 min per day in outdoor activities. Confounding factors also need to be considered. Student with one or both myopic parents were 2.86 and 3.80, respectively more likely to become myopic than those whose parents were non-myopic (Table 7). The prediction accuracy of this analytical model was 75%.

**Table 7:** Binary multi-factor logistic regression of eye behavior, parents’ myopia and students’ screening myopia obtained from the questionnaire survey.

Independent variable	OR (95% CI)	*P*
Average total time for near work per day		
<180 min	1.00	
≥180 min	3.12 (1.53–6.35)	0.002
Average total time for outdoor activities per day		
≥90 min	1.00	
<90 min	2.61 (1.29–5.26)	0.008
Parents		
No myopic parent	1.00	
One myopic parent	2.86 (1.19–6.87)	0.019
Two myopic parents	3.80 (1.50–9.60)	0.005

*P* value represents the binary multi-factor logistic regression analysis of eye behavior and parental myopia obtained from the questionnaire survey.

## Discussion

Ocular structures continue to develop during early childhood. Emmetropization is the process of precise and coordinated changes in refractive components of the eye to achieve an optical perfection; and a derailed development may result in refractive errors. The refractive error results from a multifactorial condition involving a complex interplay between the cornea, the lens and the length of the eye (11, 12). Children aged 7–12 years old are in an essential stage of myopia occurrence and development (13), making them the key population in the prevention and control of myopia. Apart from the widely acknowledged genetic disposition, eye use behavior and visual environmental influence have also been frequently reported as the causes of myopia. Number of studies have provided evidence-based recommendations of an appropriate eye use behaviors for children and adolescents in an attempt to reduce occurance of myopia (14).

This study utilized three-fold investigation methods; first, it examined the relationships between refractive status of eye with its biometric parameters; second, it looked at the correlation between objective assessment vs. the subjective reporting of the study parameters; and third, it evaluated whether eye use behavior and environmental factors were associated with myopia. While survey questionnaire have been the primary means of gathering information on eye use behavior traditionally, recently, technologically advanced electronic devices are commercially available which are more efficient and accurate in data collection. Wearable sensors are increasingly used in continuously monitoring and recording the eye use behavior objectively (15). Clouclip M2 is found to be more accurate in determining the light level into the eye (16) compared to other available devices such as Actiwatch (17), Fitsight (18) and HOBO (19). The Clouclip records eye use duration and distance in real-time and continuously processes the data (20). Moreover, Clouclip not only measures the viewing distance but also records luminance (18). Number of studies have convincingly claimed that Clouclip has a good practicability, is accurate and has a good stability in the objective measurement of working distance, eye use time and luminance, for which, it is highly recommended for myopia related research studies (21, 22).

It is worth noting that, in previous studies, the correlation between SE and ACD has been inconsistent. For instance, consistent with the result of Hosny (22), Wang et al. (24) found a negative correlation (*r* = −0.623, *P* < 0.01), between ACD and SE in children aged 5–12 years in Lanzhou (*r* = −0.498, *P* < 0.01), while Zhou et al. (25) reported no significant correlation in children aged 3–14 years. Such varying results may be related to the differences in race, region, age range, refractive status, and ocular biometrics of the subjects studied. AL and CR are the two primary parameters governing the refractive status of the eye. The ratio of axial length to corneal curvature (AL/CR), first proposed by Grosvenor (26), has been frequently used in predicting onset and monitoring myopia progression where cycloplegic refraction is not available (27). The AL/CR > 3.0 is proposed to indicate myopia (28). This ratio, being an objective measure, is relatively more reliable for less interference by subjective and regulatory factors. Consistently, our results showed that SE was highly correlated with AL/CR (*r* = −0.859, *P* < 0.01).

Compared to the subjective estimate using survey questionnaire, the Clouclip M2 measurements yielded significantly longer average total time for near work per day, the maximum time for single near work and the average distance of near work (*P* ≤ 0.001), and significantly shorter average total time for outdoor activities per day (*P* < 0.001). There are two possible reasons for this difference. Firstly, the questionnaire survey is influenced by the respondents’ subjective estimate and memory. Parents are likely to believe that myopia is caused by individual factors such as improper eye posture, and therefore may have responded with their pre-meditated “mindset”. This was evident in our result where study time was negatively associated with outdoor activity time. The study time of students in this study primarily determined by the homework set by teachers and extra study tasks set by parents. Secondly, parents and students needed to remember the time while responding the questionnaires with possibility of recall bias. Study time, outdoor time, and near work distance are mere estimates, and such bias is inevitable. Clouclip device allows a real-time recording objectively potentially eliminating the subjective bias. Accuracy in reported time may have significant implications in interpreting the results.

Duration of near-work and shorter working distance have been associated with increased risk of myopia development and progression (29, 30). However, whether these have a casual effect or this is by a mere chance is debated. A study highlighted that children with myopia tend to engage in more near work than children without myopia (31) whereas another study found no or limited role of near work as pathogenesis of myopia (32). According to a meta-analysis, 10 out of 15 cross-sectional studies found that increased prevalence of myopia is associated with longer near-work activities (32). In this study, Clouclip measured near working distance of 32.21 cm which is slightly further than that determined subjectively using survey questionnaire (24.94 cm) but is consistent with a previous report (32 cm) for among 15 years old (33). The observed differences may be linked to variations in data sources and collection method. Previous studies have shown that students spending more than 3 h a day in close work had significantly increased AL compared to those spending less than 3 h (35). Shorter near working distance (<30 cm) and longer reading time (>30 min) increased the risk of myopia development by 2.5 times and 1.5 times (30), respectively suggesting that too long near work and too close working distance are risk factors of myopia. Considering 180 min of daily near working time, 60 min of engagement in a single near task and 30 cm of working distance as cut of values for clouclip (15, 30, 34, 35). Our results, both for Cluclip and questionnaire data, consistently found that myopia was associated with the longer time spent for near work which was evident in the result of our binary multivariate analysis. The results of questionnaire survey and clouclip showed that sustained proximity time and working distance had an effect on the incidence of myopia but were not risk factors for myopia. 30 cm of working distance was not associated with myopia as the results of clouclip.

Some earlier studies have shown that the average outdoor time measured by Clouclip was 24 min per day on weekdays and 54 min per day on rest days (36), which was lower than the value in this study (61.50 min). The difference may be attributed to the variation in geographic regions, season in which the study was conducted and student articipants’ learning intensity. Previous studies have suggested that increasing outdoor activities may effectively reduce myopia occurrence in school-age children (31, 37). Results of this study, obtained from both survey questionnaire and Clouclip, consistently showed that children spending outdoor for more than 90 min per day were significantly less likely to develop myopia. Our results suggest that students with less than 200 lux daytime luminance were 2.04 times more likely to develop myopia which supports previous proposal of outdoor activity duration and luminance influencing myopia (38). These results may support earlier hypothesis that exposure to higher-intensity daylight may prevent the onset of myopia in children (19, 39).

An item in our survey questionnaire contained information on whether they use eye protection lamp as a measure to for myopia control. The results revealed that the proportion of myopia was significantly higher among those students using eye protection lamps. This finding contradicts a general belief that the lamp should have controlled myopia. Although we did not ask whether they started using the protection lamp prior to or after the onset of myopia, the unexpected finding could be related to the fact that parents of already myopic students were more concerned to their children's refractive error and they replaced their standard light with the lamp in an attempt to arrest myopia from further progression. Further investigation is required to accurately determine the protective or causative effect of the protection lamp on myopia.

Parental myopia is widely known to influence myopia occurrence in children. A previous study from Qingdao, China, found that children with both myopic parents were 2.58 times more likely to develop myopia compared to those without myopic parents (40). Consistent with the report, we found that children with one myopic parent were 2.86 times likely to become myopic. Children who have both myopic parents are 3.80 times higher chance of becoming myopic compared to those without any myopic parent. These results further supports the arguably undeniable role of genetic factors in the incidence of myopia in children.

Our study has some limitations. Although the Clouclip M2 measures luminance with a reasonable accuracy, it does not detect the type of light source (e.g., natural light vs. electric light). Also, it does not identify whether the wearer is looking at paper surface or monitor of an electronic device. Although it is yet to be determined whether above mentioned factors have any influence on myopia development, it would be desirable to investigate in the future. Therefore, studies of this nature should use an appropriate questionnaire tool along with the device to monitor associated factors more accurately. Further, our data suggest that myopic children were more compliant with study procedure and wore the device in a standardized manner compared to non-myopic children during the study. However, the difference in compliance was practically insignificant; therefore, we believe that the difference would have none to minimal influence in the overall outcome of this study. The relatively smaller sample size of non-myopic students as compared myopic sample is another limitation of the study. Future study with more balanced sample size is desirable for better credibility of the results.

In conclusion, our study further supports the proposal of eye-using behavior, eye-using environment, and parents’ myopia are strongly associated in the occurrence and development of myopia in children. Clouclip M2 provides valuable information on eye use behavior and visual environment which can be used in investigating myopia related studies. Addition of more elaborative items in survey questionnaires in relation to specific eye use behaviors may be useful in better understanding of the effect of environmental factors on development of myopia.

## Data Availability

The original contributions presented in the study are included in the article/**Supplementary Material**, further inquiries can be directed to the corresponding author.
